# Diabetic ketoacidosis among adult patients with diabetes mellitus admitted to emergency unit of Hawassa university comprehensive specialized hospital

**DOI:** 10.1186/s13104-019-4186-3

**Published:** 2019-03-14

**Authors:** Asres Bedaso, Zewdie Oltaye, Ephrem Geja, Mohammed Ayalew

**Affiliations:** College of Medicine and Health Sciences, School of Nursing, Hawassa University, P.O.BOX: 1560, Hawassa, SNNPR Ethiopia

**Keywords:** Diabetic ketoacidosis, Diabetes, Hawassa, Prevalence

## Abstract

**Objective:**

This study was aimed to assess the prevalence and associated factors of diabetic ketoacidosis among adult patients admitted in emergency department of Hawassa university comprehensive specialized hospital. An institution based retrospective cross-sectional study design was conducted among 195 adult patients aged 16 years and above with known or previously unknown diabetes cases presented in the emergency unit.

**Result:**

In our study from the total 195 patients medical record reviewed 78 (40%) developed DKA. Out of the total reviewed medical record 55 (28.2%) and 23 (11.8%) were with type-1 and type 2 diabetes mellitus respectively. From acute complication of diabetes, diabetic ketoacidosis was a leading cause 78 (77%) followed by hypoglycemia 14 (14%) and hyperosmolar hyperglycemic state (9%). During multiple logistic regression analysis age and hypertension were found to have significant association with diabetic ketoacidosis.

**Electronic supplementary material:**

The online version of this article (10.1186/s13104-019-4186-3) contains supplementary material, which is available to authorized users.

## Introduction

Diabetes is a group of metabolic disorder characterized by hyperglycemia resulting from defects in insulin secretion, insulin action or both [1]. Diabetes mellitus DM) is a group of common metabolic disorders that share the phenotype of hyperglycemia, which are caused by a complex interaction of genetics and environmental factors. It is the leading cause of end-stage renal disease, traumatic lower extremity amputations, and adult blindness. In Ethiopia study shows the prevalence of DKA among acute complication of DM was 68.3% and 71% in Dessie and Jimma respectively [2].

The major classifications of diabetes are type-1 diabetes which is caused by β-cell destruction and type-2 diabetes that results from insulin resistance. Type-1 DM covers 5–10% of all diabetes and type-2 DM covers 90–95% of all diabetes [3]. Diabetes and its complications are major causes of early death in most countries, with cardiovascular disease being the leading cause of death among people with diabetes [4].

Diabetes mellitus leads to acute and chronic complications include diabetic ketoacidosis (DKA), hyperosmolar hyperglycemic state, and hypoglycemia during treatment. Diabetic ketoacidosis (DKA) is one of the most serious acute complications of DM. Ketoacidosis occurs when stored triglyceride broken down into fatty acid which serves as alternate sources of fuel, which causes elevation of blood ketone leads to ketoacidosis [5].

The common causes of DKA are missed dose of insulin, illness or infection, and undiagnosed or untreated diabetes. The main clinical features of DKA are hyperglycemia, dehydration, electrolyte loss, and acidosis [6]. Diabetic ketoacidosis (DKA) occurs commonly in people who have type 1 diabetes. However, people who have type 2 diabetes may also develop diabetic ketoacidosis [7].

In Africa, 19.8 million people or 4.9% are estimated to have diabetes in 2013 [8]. In Ethiopia, WHO estimates the number of cases of diabetics to be about 800,000 in 2000 and projected that it would increase to about 1.8 million by the year 2030 [9]. The same studies conducted in Ethiopia from 1970 to 2011 suggested that DM prevalence in the country was about 2%, rising to > 5% in persons aged ≥ 40 years in certain settings [10]. A more recent nationwide World Health Organization (WHO) Steps survey among 2153 persons in Ethiopia found the DM prevalence to be 6.5% [11].

DKA is one of the most fatal acute complications among DM patient. Its mortality rate ranges from 2 to 5 percent in developed countries and 6 to 24 percent in developing countries. If it misdiagnosed or mistreated, it is 100% fatal [12]. In some studies it has been reported that DKA can be present in 25% to 30% of type 1 diabetes cases at onset and from 4 to 29% in youth with type-2 diabetes [13].

Several studies were conducted on diabetes mellitus and its complication, but few studies were done on the prevalence and factors associated with DKA among Ethiopian patients. As a result of this information’s are scant to promote better health service to prevent mortality due to DKA in Ethiopia.

## Main text

### Methods and materials

#### Study design, area and period

Institution based retrospective cross-sectional study design was conducted at Hawassa university comprehensive specialized hospital from February 01–30/2018. Hawassa is the capital city of SNNPR of Ethiopia, which is 273 km far from South of Addis Ababa. The hospital gives service for about 18 million people of SNNPR and neighboring areas of Oromia regional state. It has 350 beds for admitted patients. The hospital provides different service through inpatient and outpatient level. The hospital’s emergency unit gives services for approximately 10,000 patients per year. The emergency unit has more than 33 beds and 10 rooms for emergency admission.

#### Population

All adult DM patients who visited Hawassa university comprehensive specialized hospital emergency unit from January, 2016 to January, 2018 were the source population. Diabetes patients, who visited adult emergency unit of Hawassa university comprehensive specialized hospital from January, 2016 to January, 2018 and those who fulfill the inclusion criteria were the study population.

#### Sample size and sampling technique

Single population proportion formula was used to calculate sample size with the assumption of, 5% margin of error, 95% confidence interval and 71% proportion of DKA from a study conducted in Jimma University teaching hospital [2]. Since the source population was < 10,000, we used a correction formula, adding 10% non response rate, the final sample size was 195.

Systematic random sampling technique was used to select the record of the study subjects. The record of the study subjects were selected based on constant interval K = 2 calculated from total DM case admitted at emergency unit from 1/2016 to 1/2018 in chronological order, which were 400 divided by the sample size of the study 195. The first patient was selected by a lottery method with in the first two medical record number and all patient charts in K interval was included in the study until the calculated sample size was obtained.

#### Data collection instrument and data collection technique

A structured and adapted questionnaire was used and pre-test was conducted in Adare hospital 5% of the sample size. The data was collected using structured questionnaire. The collected data were entered and analyzed using SPSS version 20. Binary and multiple logistic regression analysis was used to see the association between outcome and explanatory variables. During multiple logistic regression, the strength of association was measured by odds ratio with 95%CI and P-value less than 0.05 was considered as statistically significant.

#### Operational definition

Short-acting insulin treat: 30 min–1 h of onset (length of time before insulin reaches bloodstream).

Intermediate acting: 1.5–4 h of onset (length of time before insulin reaches bloodstream).

Long-acting: 0.8–4 h of onset (length of time before insulin reaches bloodstream).

### Results

#### Socio demographic characteristics

There were total of 400 adult patients who were admitted with DM in emergency room of Hawassa university comprehensive specialized referral hospital during two years period (January/2016–January/2018). Of these 400 patients, 195 diabetic cases were reviewed. 125 (64.1%) of the study subjects were male and 52 (26.7%) were within the age group of 25–34 (Table 1).

**Table 1:** Socio demographic characteristics of DM patient visited to Hawassa university comprehensive specialized hospital emergency unit from 2016 to 2018 (n = 195)

Variables	Characteristics	N	%
Age	15–24	28	14.4
	25–34	52	26.7
	35–44	49	25.1
	45–54	22	11.3
	≥ 55 years	44	22.6
Sex	Male	125	64.1
	Female	70	35.9
Residence	Urban	100	51.3
	Rural	95	48.7
Health insurances	Insured	11	5.6
	Non insured	184	94.4
Referred by	Self	119	61
	Health center/hospital	76	39
Hypertension	Yes	42	21.5%
	No	153	78.5%

#### Prevalence of diabetic ketoacidosis among patients with DM

Among the reviewed 195 diabetic patients medical record, 114 (58.46) were type 1 DM and 81 (41.54%) were type 2 DM. From the total 195 patients, type-1 DM with DKA was 55 (28.2%) and type-2 DM with DKA was 23 (11.8%) (Table 2).

**Table 2:** Prevalence of DKA patient visited to Hawassa university comprehensive specialized hospital emergency room from January 2016 to January 2018 GC

DM type	Frequency			Percent		
	Male	Female	Total	Male	Female	Total
Type 1 with DKA	35	20	55	17.9	10.2	28.2
Type 1 without DKA	41	18	59	21	9.2	30.3
Type 2 with DKA	17	6	23	8.7	3	11.8
Type 2 without DKA	32	26	58	16.4	13.34	29.7
Total	125	70	195	64.1	35.89	100

Also from the total reviewed 195 records, 128 (65.6%) were known DM and 67 (34.4%) were newly diagnosed, among them, known DM with DKA were 36 (46.15%) and newly diagnosed DM cases with DKA were 42 (53.85%) (Additional file 1).

#### Acute and chronic complications of DM

Among acute complication of DM, DKA was the leading causes 78 (77%), followed by hypoglycemia 14 (14%), and hyperosmolar hyperglycemic state (HHS) 9 (9%). The most common chronic complication was DFU (diabetic foot ulcer) 60%, followed by retinopathy 27%, and nephropathy 13%.

#### Anti diabetic medication profile

Most DKA patients 59 (75.64%), commonly taking short acting agents, followed by intermediate acting 19 (24.36%) (Additional file 2).

#### Sign and symptom of DKA/DM/

In this study the frequently reported presenting symptoms of DKA or DM were 3P (polydipsia, polyuria, polyphagia) (50.3%), followed by 2P (polyuria and polydipsia) (29.2%), others (15.9%), and weight loss 1.5%.

#### Factors associated with DKA

During multiple logistic regression analysis age, duration of DM with DKA and hypertension, found to the significant factors for developing DKA. Age groups between 15–24 and 25–34 years and HTN have significant association with developing DKA among patients (Table 3).

**Table 3:** Factors associated with diabetic ketoacidosis among patient visited to Hawassa university comprehensive specialized hospital emergency room from January 2016 to January 2018 GC

Variable	Category	DKA		COR (95%)	AOR (95% CI)
		Yes	No		
Age	15–24	17	11	0.19 (0.068, − 0.536)	0.21 (0.072, 0.60)*
	25–34	31	21	0.199 (0.081, − 0.488)	0.22 (0.087, 0.54)*
	35–44	15	34	0.667 (0.263, − 1.691)	0.69 (0.27, 1.77)
	45–54	5	17	1.000 (0.295, 3.391)	1.03 (0.302, 3.523)
	≥ 55	10	34	Ref.	Ref.
Residence	Urban	34	66	1.675 (0.94, 2.984)	1.29 (0.688, 2.42)
	Rural	44	51	Ref.	
HTN	Yes	7	35	0.231 (0.097, 0.552)	2.78 (1.095, 7.074)*
	No	91	62	Ref.	

*COR* crudes odds ratio, *AOR* adjusted odds ratio, *CI* confidence interval

* Significant Association with P value < 0.05

### Discussion

The prevalence of diabetic ketoacidosis among DM patients in this study was 40%. It is close to former studies conducted in Italy (40.3%) [14] and Collared university (38.6%) [12]. However, low prevalence has been reported in the a previous studies conducted in Cambridge University (18–22%) [6], Nigeria (12.2%) [4], and England (22.5–23.9%). The variation might be due to the socio-cultural differences in health seeking behavior, a change in feeding and overall life style emanating from increasing urbanization and economic development in the region.

In this study the prevalence of DKA in type 1 DM and type 2 DM was 28.7% and 11.28% respectively. The current finding was in line with studies conducted in Colorado American university [13], prevalence of DKA in type one diabetes and type 2 was 25–30% and 4–29% respectively.

In our study, among newly diagnosed DM case, 53.85% of cases present with DKA. This figure is more than what observed in England (22.5 to 23.9%) [15]. The variation might be due to socioeconomic status, race/ethnicity variation.

In the current study the prevalence of DKA among acute complications of diabetes is 77%. The finding was higher than the previous studies done in Dessie (68.3%) [2]. The variation might be due to the different study design used and different socio-demographic characteristics of clients.

In the study younger age was significantly associated with DKA, the finding was in line with studies conducted in Jima referral hospital [2], Colorado university [13] and findings in four countries England, Wales, Austria, US and Germany [16]. Another study conducted in Cambridge university indicated that age had significantly associated with diabetic ketoacidosis [6].

In this study being male, and being younger age group were significantly associated with DKA, similar to previous finding in Italy [14]. Ketosis-prone diabetes mellitus (KPDM ) was more frequent in men compared to women and in obese compared to non-obese participants. Gender differences in KPDM may be associated with a different impact of obesity in men and women. Although multiple factors (e.g., ethnic minority, lack of health insurance, lower body mass index, preceding infection, delayed treatment) affect the risk of developing DKA among children and young adults, intervention is possible between symptom onset and development of DKA [6].

### Conclusion

The prevalence of DKA among diabetes observed in this study was 40%. The study found that diabetic ketoacidosis is more common in male and young adults’ particular between the ages of 25–34 years.

## Limitation

Since the study is cross sectional, it cannot identify cause and effect relationship.

## Additional files

**Additional file 1.** Profile of patients Type-1 and Type-2 diabetes with DKA among patient visited to Hawassa university comprehensive specialized hospital emergency room from January 2016 to January 2018 GC.

**Additional file 2.** Anti diabetic medication profile of patients T1 & T2 diabetes with DKA versus without DKA among patient visited to Hawassa university comprehensive specialized hospital emergency room from January 2016 to January 2018 GC.

## Abbreviations

AOR: adjusted odds ratio
CI: confidence interval
COR: crudes odds ration
DFU: diabetic foot ulcer
DKA: diabetic ketoacidosis
DM: diabetes mellitus
GC: Gregorian calendar
HHS: hyperosmolar hyperglycemic state
HTN: hypertension
KPDM: ketosis prone diabetes mellitus
MRN: medical record number
SNNPR: Southern Nations Nationalities and Peoples Region
US: United States
WHO: World Health Organization
