# Supplementary material for: Diabetic ketoacidosis among adult patients with diabetes mellitus admitted to emergency unit of Hawassa university comprehensive specialized hospital
Source: BMC Res Notes. 2019 Mar 14;12:137. doi: 10.1186/s13104-019-4186-3 (PMC6419397; doi:10.1186/s13104-019-4186-3)
Supplement: Supplementary file 1 — Additional file 1. Profile of patients Type-1 and Type-2 diabetes with DKA among patient visited to Hawassa university comprehensive specialized hospital emergency room from January 2016 to January 2018 GC. [file 13104_2019_4186_MOESM1_ESM.docx]

**Additional file 1:** Profile of patients Type-1 and Type-2 diabetes with DKA among patient visited to Hawassa university comprehensive specialized hospital emergency room from January 2016 to January 2018 GC.

| **Variable** | **Type 1 with DKA** | | **Type 2 with DKA** | | **Total** | |
| --- | --- | --- | --- | --- | --- | --- |
|  | **N** | **%** | **N** | **%** | **N** | **%** |
| Known DM | 26 | 33.34 | 10 | 12.82 | 36 | 46.15 |
| Newly diagnosed DM | 30 | 38.46 | 12 | 15.38 | 42 | 53.85 |
| Total | 56 | 71.79 | 22 | 28.2 | 78 | 100 |
