# Supplementary material for: Diabetic ketoacidosis among adult patients with diabetes mellitus admitted to emergency unit of Hawassa university comprehensive specialized hospital
Source: BMC Res Notes. 2019 Mar 14;12:137. doi: 10.1186/s13104-019-4186-3 (PMC6419397; doi:10.1186/s13104-019-4186-3)
Supplement: Supplementary file 2 — Additional file 2. Anti diabetic medication profile of patients T1 & T2 diabetes with DKA versus without DKA among patient visited to Hawassa university comprehensive specialized hospital emergency room from January 2016 to January 2018 GC. [file 13104_2019_4186_MOESM2_ESM.docx]

**Additional file 2**: Anti diabetic medication profile of patients T1 & T2 diabetes with DKA versus without DKA among patient visited to Hawassa university comprehensive specialized hospital emergency room from January 2016 to January 2018 GC.

| **Type of insulin administered** | **DM Type (N=195)** | | | | **Total** |
| --- | --- | --- | --- | --- | --- |
|  | **Type 1 with DKA** | **Type 1without DKA** | **Type 2 with DKA** | **Type 2 without DKA** |  |
| Short acting | 45 | 30 | 14 | 21 | 124 |
| Intermediate acting | 10 | 15 | 9 | 23 | 59 |
| Long acting | 0 | 1 | 0 | 5 | 6 |
| Others | 0 | 13 | 0 | 9 | 6 |
| Total | 55 | 59 | 23 | 58 | 195 |
